# FEMOSEAL CLOSE: multi-centre observational study with FemoSeal™ vascular closure device following peripheral percutaneous endovascular procedures

**DOI:** 10.1186/s42155-025-00522-5

**Published:** 2025-02-22

**Authors:** Yann Gouëffic, Koen Deloose, Maxime Dubosq, Thomas Zeller

**Affiliations:** 1 Department of Vascular and Endovascular Surgery, Groupe hospitalier Paris St Joseph, Paris, France; 2 Department of Vascular Surgery AZSint Hospital Dendermonde, Dendermonde, Belgium; 3 Aortic Centre, Institut Coeur-Poumon, CHU Lille, Lille, France; 4 Department Angiology, University-Heart Center Freiburg, Bad Krozingen, Germany

**Keywords:** Vascular closure device, Peripheral arterial disease, Endovascular

## Abstract

**Background:**

The purpose of the FEMOSEAL CLOSE study was to assess the safety and effectiveness of FemoSeal™ vascular closure device in achieving haemostasis following transfemoral peripheral procedures in routine clinical practice.

**Materials and methods:**

This prospective, European, multi-centre, single-arm, post-market clinical follow-up study enrolled patients undergoing diagnostic or interventional peripheral endovascular procedures with FemoSeal™ vascular closure device in inpatient or outpatient settings. The primary endpoint was a composite of safety and effectiveness, with effectiveness defined as cessation of arterial bleeding without adjunctive treatment, and safety as freedom from major vascular complications in the first 6 h. Secondary endpoints included: minor access-site complications and major complications 6 h to 30 days post-procedure, and time to haemostasis, ambulation and discharge. Quality of life was assessed at baseline, discharge and 30-day follow-up. Device usability was also surveyed.

**Results:**

Between December 2021 and July 2022, 230 patients were enrolled in three European centres. The primary composite endpoint was achieved in 95.1% (215/226) [95% confidence interval: 91.46–97.55] of patients. The effectiveness component was achieved in 96.9% (219/226) [95% confidence interval: 93.70–98.70], and the safety component in 95.2% (220/230) [95% confidence interval: 92.15–97.90] of patients. FemoSeal™ vascular closure device deployment failure occurred in 1.6% (4/230) of cases, with subsequent manual compression achieving haemostasis. The median time to haemostasis was 0.42 min.

**Conclusions:**

The study device provides effective haemostasis and low rates of access-site complications up to 30 days post-procedure for patients undergoing peripheral endovascular interventions. It demonstrates good performance with rapid haemostasis.

**Trial registration:**

The study is registered at clinicaltrials.gov. (ClinicalTrials identifier: NCT05027698).

**Supplementary Information:**

The online version contains supplementary material available at 10.1186/s42155-025-00522-5.

## Background

Nowadays, many patients undergo percutaneous endovascular procedures worldwide for various coronary, peripheral and neurovascular interventions. The arterial access puncture site can be closed post-procedure by manual compression (MC) or via a vascular closure device (VCD). Compared with MC, VCDs reduce time to haemostasis (TTH) [1, 2], ambulation and discharge [2], with comparable rates of vascular complications [2].

FemoSeal™ VCD (Terumo Corp., Japan) achieves haemostasis by sealing the access puncture site between two bioabsorbable discs. The study device has been evaluated in two large randomised controlled studies of patients undergoing coronary angiography (CAG) via femoral access [3, 4]. In both studies, the study device achieved haemostasis more rapidly than MC, with comparable safety [3, 4]. The study device has also shown good efficacy and safety in patients undergoing peripheral arterial disease (PAD) endovascular revascularisation and superiority in procedural technical success to a suture-based VCD (ProGlide™, Abbott, USA) [5]. 

The positive profile of the study device in terms of its efficacy and safety is supported by registry data [6, 7]; however, real-world evidence, especially in patients undergoing PAD endovascular procedures, is needed. Our study assesses the safety and effectiveness of the study device in achieving haemostasis following PAD endovascular procedures performed via common femoral artery (CFA) access. The study device was used according to the instruction for use (IFU) [8], as part of the post-market clinical follow-up surveillance plan in this real-world prospective study.

## Materials & methods

### Study design and patients

This multi-centre, prospective observational study was conducted at three European centres (Belgium, France and Germany) in patients undergoing diagnostic or interventional peripheral endovascular procedures using FemoSeal™ VCD.

Patient inclusion and exclusion criteria are presented in Tables 1. Patients meeting one of the exclusion criteria were considered screening failures and followed up until discharge; remaining patients were followed up at 30 days (± 7 days) with a hospital visit or telephone call (if this is not standard clinical practice).

**Table 1:** Study inclusion and exclusion criteria

Inclusion criteria	Exclusion criteria
• ≥ 18 years old • Undergoing diagnostic or interventional endovascular procedure compatible with the use of the study device • Puncture site located at the CFA (i.e. between the inferior epigastric artery and the bifurcation of the superficial and profunda femoral arteries) • The study device deployed as per IFU by a trained operator on arteriotomy where sheaths or devices of ≤ 7 F were used, following a femoral artery angiogram	• Any contraindication to the endovascular treatment and/or the study device use as per IFU • Use of the study device on puncture sites other than the CFA • Re-puncture of the CFA within 90 days at the same access site • Enrollment of a patient with the contralateral CFA puncture, when a prior target-limb access site has been selected for the study and a FemoSeal™ has been deployed Additional intra-operative exclusion criteria: • Lumen diameter of the CFA < 5 mm • Stenosis and/or significant plaque present in the vicinity of the CFA puncture site • Arterial puncture is at, or distal to, the CFA bifurcation • Anomalous branches or vessel abnormalities present in the vicinity of the CFA puncture site • Use of > 7 F primary introducer sheaths or devices • Multiple femoral punctures • Known or suspected posterior femoral wall puncture • Any condition that would make use of the closure device inappropriate (as per investigators’ discretion)

The study was conducted in compliance with Good Clinical Practice, the Declaration of Helsinki, ISO 14,155 and MDR (EU) 2017/745, and approved by local medical ethics committees.

### Procedures

Patients underwent planned diagnostic or interventional endovascular procedures with the study device using a ≤ 7 F (2.33 mm) procedural sheath. If standard of care, a retrograde or antegrade CFA puncture was performed under duplex US (DUS) guidance. Otherwise, the puncture site was assessed with femoral angiography before the study device deployment, and deployment discontinued if one of the intra-operative exclusion criteria was identified (Table 1).

The study device was deployed as per the IFU [8]. The VCD is deployed over an exchange wire after removal of the standard vascular access sheath. Using a single-push button, the inner and outer discs move to sandwich and seal the puncture site on the inner and outer arterial surfaces. After VCD removal, the suture is cut under skin level to complete the closure.

Patients received medication according to the hospital’s standard of care. At 30-day follow-up, the following information was collected: access-site complications and pain, DUS of closure site (patients attending hospital only), EQ-5D questionnaire [9–11], use of concomitant antiplatelet and/or oral anticoagulant medication, and adverse events.

### Outcome measures

The primary endpoint was a composite of safety and effectiveness of successful FemoSeal™ puncture site haemostasis and freedom from major complications of the access-site limb within 6 h post-procedure in patients in whom the study device was successfully deployed. The effectiveness component of successful puncture site haemostasis was defined as cessation of arterial bleeding (excluding oozing) achieved in the CathLab in patients not requiring any adjunctive intervention, including MC and/or wound dressing. Oozing was defined as diffuse capillary haemorrhage, without pulsatile flow, not causing haematoma enlargement. Major complications were defined as access site: vascular injury requiring surgical or endovascular repair, bleeding (type 2, 3 or 5 Bleeding Academic Research Consortium [BARC] classification) following study device deployment, any ipsilateral acute leg ischaemia attributable to the study device (documented by patient’s symptoms, physical exam and/or imaging), or major infection (i.e. requiring intravenous antibiotics and/or extended hospitalisation).

Secondary endpoints included: freedom from minor complications at the access site within 6 h post-procedure, freedom from major and minor complications at the access site 6 h to 30 days post-procedure, TTH, time to ambulation (TTA), length of hospital stay (LoS), quality of life (QoL) assessed by the EQ-5D questionnaire [9–11]. The QoL assessment did not ascribe any reasons for changes in QoL. A subjective assessment of the study device usability via operator questionnaire was also performed.

### Statistical analysis

**Fig. 1:**
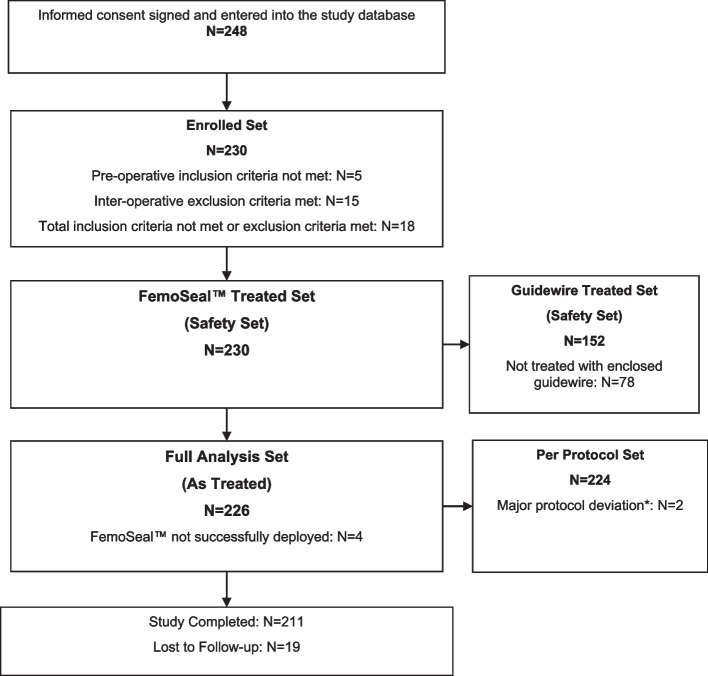
Study flowchart. *Major deviations included problems with informed consent, exclusion/inclusion criteria met/not met (except screening failure), device not used according to instructions for use

Safety was analysed in a FemoSeal™-treated analysis set including all enrolled patients who met the inclusion and exclusion criteria, in whom the study device use was attempted, regardless of deployment success (Fig. 1). Effectiveness was analysed in the full analysis set (FAS) that included patients in whom the study device was successfully deployed. The primary composite endpoint was reported for the FAS.

The percentage of patients who successfully received the study device is presented with 95% confidence interval (CI) representing the likely range of this percentage in the real world. Similar methods were used to analyse endpoints reporting freedom from complications at 6 h post-procedure and at 30-day follow-up. Proportional hazards model was used to evaluate TTH, TTA and LoS.

A *post hoc* analysis studied freedom from minor and major adverse events from the moment of study device deployment until 30 days after the procedure.

QoL data from EQ-5D were reported as absolute values and change from baseline in each domain; EQ-VAS and the health state value were reported by visit. The Wilcoxon signed-rank test was used to describe the significance of change from baseline to hospital discharge and to 30-day follow-up.

An ad hoc subgroup analysis compared TTH, TTA, LoS and complication rate for sex, body mass index (BMI < 30 kg/m^2^ or ≥ 30 kg/m^2^), puncture type (antegrade/retrograde), or with/without DUS. These subgroups were investigated for dichotomous endpoints by logistic regression model with odds for each subgroup level presented. The model was fitted separately for each subgroup with the odds ratio and 95% CI. For time-to-event endpoints, Cox proportional hazard models were fitted. An ANOVA was presented with Type III tests and p-values representing the unique contribution the covariate makes to the model.

## Results

Data for 248 patients were entered into the study database, of these 18 were screen failures. The device was deployed in two screen failures; a bleeding event (BARC criteria II) occurred in one screen failure, and this was resolved using MC.

The remaining 230 patients met all inclusion and exclusion criteria and were included in the FemoSeal™-treated set. The device was not successfully deployed in 4 of these patients leaving 226 patients qualifying for the FAS.

### Baseline characteristics

A total of 230 patients met the inclusion and exclusion criteria and were enrolled: 70% (161) were male, mean ± SD age was 70.9 ± 11.69 years, and median BMI was 26.1 ± 4.95 kg/m^2^ (Table 2). At baseline, 211 (91.7%) patients received antiplatelet therapy; of them, 21 (9.1%) received dual antiplatelet therapy. Oral anticoagulation was prescribed for 40 (17.4%) patients.

**Table 2:** Baseline patient characteristics and medical history

Patient baseline characteristics	*N* = 230, n (%)^a^
Age, y (mean ± SD)	70.9 ± 11.69
Age, median (min; max)	72.0 (63; 79)
Gender, male	161 (70)
Body mass index, kg/m^2^ (mean ± SD)	26.1 ± 4.95
Diabetes mellitus	72 (31.3)
Hypertension	160 (69.6)
Chronic kidney disease	30 (13)
Liver cirrhosis with portal hypertension	1 (0.4)
Chronic obstructive pulmonary disease	19 (8.3)
Coronary artery disease	42 (18.3)
Congestive heart failure	11 (4.8)
Dyslipidaemia	128 (55.7)
Atrial fibrillation	22 (9.6)
Carotid artery disease	21 (9.1)
Anaemia	10 (4.3)
Smoking	
• Current	86 (37.6)
• Previous smoker	66 (28.8)
*Comorbidities*	
Coronary revascularization	34 (14.8)
Myocardial infarction	18 (7.8)
TIA/Stroke	27 (11.7)
Carotid artery revascularization	12 (5.2)
• Carotid artery endarterectomy	1 (0.4)
• Carotid artery stenting	11 (4.8)
Lower-limb revascularization	82 (37.5)
• Low-limb surgical bypass	8 (3.5)
• Percutaneous intervention	74 (32.2)
Coagulation disorder	1 (0.4)

*TIA *transient ischemic attack,* SD *standard deviation

^a^*N *= number in the analysis set, *n* = number of patients, %=(n/non-missing N)×100

Procedures were performed in inpatient and outpatient settings (70.4% and 29.6%, respectively). The main procedural indication was claudication (Table 3). Femoral access was achieved by antegrade (15.3%) or retrograde puncture (84.7%). DUS could be used to guide both puncture and/or closure; the use of DUS was at the operator’s discretion: DUS-guided puncture was used in 105 patients (45.7%) while DUS-guided closure was employed in 87 (37.8%) of patients.

**Table 3:** Procedural characteristics and unsuccessful deployment

Procedural characteristics	*N* = 230, n (%)^a^
Indication for endovascular procedure	
• Claudication	147 (63.9)
• Critical limb ischaemia	67 (29.1)
• In stent restenosis	4 (1.7)
• Other	12 (5.2)
Access puncture type	
• Antegrade puncture	35 (15.3)
• Retrograde puncture	194 (84.7)
DUS - guided puncture	
• No DUS	125 (54.3)
• DUS	105 (45.7)
DUS – guided VCD closure	
• No DUS	143 (62.2)
• DUS	87 (37.8)
Sheath Size (FR)	
• 5 F	4 (1.7)
• 6 F	216 (93.9)
• 7 F	10 (4.3)
Length of sheath mean (SD, cm)	44.8 (18.30)
Unsuccessful deployment:	4 (1.6)
Reasons for unsuccessful deployment:	
• The anchor did not seal	1 (0.4)
• Unable to deploy	1 (0.4)
• Device slipped through the vessel wall	2 (0.8)

*DUS *duplex ultrasound, *SD * standard deviation

^a^*N* = number in the analysis set, *n* = number of patients, %=(n/non-missing N)×100

FemoSeal™ deployment was unsuccessful in 4/230 (1.7%) cases, with subsequent MC sufficient to achieve haemostasis. In cases of device failure or incomplete haemostasis, no interventional treatment or surgical repair for acute ipsilateral leg ischaemia or haematoma evacuation was required.

Follow-up was conducted at a hospital visit in 163 (78.7%) patients and via phone call in 44 (21.4%) patients.

### Primary endpoints

The primary endpoint was achieved in 215/226 (95.1%) (95% CI: 91.46–97.55) patients (Table 4). The effectiveness component was achieved in 219/226 (96.9%) (95% CI: 93.70–98.70) patients and the safety component in 220/230 (95.2%) (95% CI: 92.15–97.90) patients.

**Table 4:** Primary and secondary endpoints

**Primary endpoints**		
	**Events (n/N)**	**% [95% CI]**
Combined safety and effectiveness endpoint in the Full Analysis Set	215/226	95.1 [91.46–97.55]
Effectiveness defined as cessation of arterial bleeding (excluding oozing) achieved in CathLab in patients not requiring any adjunctive intervention at the access site	219/226	96.9 [93.70–98.70]
Safety defined as freedom from major complications of the access-site limb within 6 h post-procedure • Major complications defined as: ◦ Vascular injury attributable to the study device that requires surgical or endovascular repair ◦ Access site-related bleeding (type 2, 3 or 5 bleeding, following study device deployment, as per BARC classification) ◦ Any new access site-related ipsilateral acute leg ischaemia attributable to the study device (documented by patient’s symptoms, physical exam and/or imaging) ◦ Access site major infection (i.e. requiring intravenous antibiotics and/or extended hospitalisation)	220/230	95.2 [92.15–97.90]
Major adverse events within 6 h of procedure:		
• Access site-related bleeding (BARC type 2, 3 or 5)* • Femoral puncture site-related hematoma (< 5 cm)	9/230 2/230	3.9 0.9
**Secondary endpoints**		
**Complications**	**Events (n/N)**	**Median [95% CI]**
Freedom from minor complications at access site for 6 h post-procedure • Minor complications defined as: ◦ Any minor (< 5 cm) or major (≥ 5 cm) femoral puncture site-related haematoma (i.e. a palpable groin swelling measured at the longest diameter) ◦ Pseudoaneurysms attributable to FemoSeal™ ◦ Femoral access site arteriovenous fistulas ◦ Minor femoral access site infections (i.e. all infections not defined as major)	225/230	97.8% [95.00; 99.29]
Freedom from major and minor complications at access site from 6 h to 30 days post-procedure	200/211	94.8% [90.86; 97.37]
Freedom from major and minor complications at access site from 0 h to 30 days post-procedure	191/211	95.2% [85.74; 94.11]
Major adverse events:^a^		
• Access site-related bleeding (BARC type 2)	11/211	5.21% [2.63; 9.14]
• Repeat manual compression (i.e. performed as a result of recurrent bleeding or extensive haematoma at the femoral puncture site following patients exit from the CathLab)	2/211	0.95% [0.11; 3.38]
Minor adverse events:^b^		
• Femoral puncture site-related haematoma (< 5 cm)*	10/211	4.74% [2.30; 8.54]
• Femoral puncture site-related haematoma (≥ 5 cm)*	4/211	1.90% [0.52; 4.78]
**Time to event endpoints**	**Events (n/N)**	**Median [min; max]**
Time to haemostasis (TTH), minutes	230/230	0.42 [0.25; 0.50]
• Defined as time from procedural sheath removal to first observed cessation of arterial bleeding (excluding oozing)		
Time to ambulation (TTA), hours	229^†^/230	5.00 [4.54; 5.50]
• Defined as time between procedural sheath removal and the moment when the patient is able to autonomously stand up and walk without recurrent bleeding		
Length of stay in the hospital (LoS), hours	230/230	23.98 [22.72; 25.00]
• Defined as time between procedural sheath removal and patient discharge		

*BARC * Bleeding Academic Research Consortium, *CI * confidence interval

*All reported bleeding events were BARC type 2

†TTA for one subject censored at the time of hospital discharge

^a^Of the four patients with major haematoma: two had BARC type 2 bleeding, one had repeated manual compression

^b^Of the ten patients with minor haematoma: three had BARC type 2 bleeding, one had repeated manual compression

### Secondary endpoints

Freedom from minor access-site complications for 6 hours post-procedure was achieved in 225/230 (97.8%) (95% CI: 95.00–99.29) patients. Freedom from major and minor access-site complications from 6 hours to 30 days post-procedure was achieved in 200/211 (94.8%) (95% CI: 90.86–97.37) patients. A *post hoc* analysis showed the overall freedom from minor and major adverse events from FemoSeal™ deployment until 30 days post-procedure was achieved in 191/211 / (91.3%) [95% CI: 85.74–94.11] patients (Table 4). Most complications were minor haematomas (10 patients, 4.74%), all bleeding events were classified as BARC type 2. No instances of limb ischaemia, arterial–venous fistula or infections were associated with study device use. The study device IFU recommends using the guidewire included with the FemoSeal™ kit (GW 0.038”); 152/230 (66.1%) patients were treated with this guidewire without reported device deficiencies or adverse events. Patients who were not treated with the guidewire supplied with the FemoSeal™ kit were treated with stiff/half-stiff guidewires (specific brands were not recorded). The median TTH with the study device was 0.42 (95% CI: 0.25–0.50) minutes – i.e 25.2 s – and median TTA was 5.00 (95% CI:4.54–5.54) hours. The median LoS was 23.98 (95% CI:22.99–25.08) hours.

**Fig. 2:**
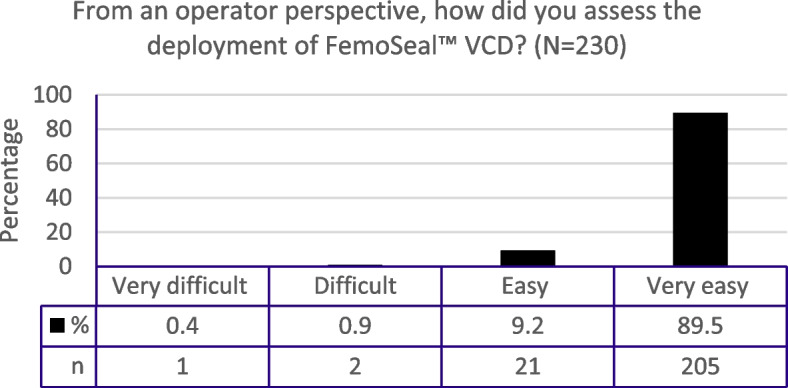
FemoSeal™ operator usability

The subjective assessment of device usability was performed, with physicians evaluating the study device as ‘easy’ or ‘very easy’ to deploy in 98.7% of cases (Fig. 2).

There was a statistically significant difference in changes in EQ-5D overall health score with an asymmetric distribution showing more patients improving than worsening, from pre-procedure to hospital discharge (*p* < 0.0001) and pre-procedure to 30-day follow-up (*p* < 0.0001) (Supplementary Fig. 1). Similar distribution patterns were observed for within-patient changes in overall health perception, assessed by visual analogue scale (VAS), from pre-procedure to hospital discharge (*p* < 0.0001) and pre-procedure to 30-day follow-up (*p* < 0.0001). These QoL changes were not ascribed to any particular aspect of the procedure or intervention.

### Subgroup analysis

There were no statistically significant differences regarding major and minor complications from VCD deployment until 30-day follow-up in any subgroup. There were no significant differences in TTH, TTA or LoS between male and female patients or between obese and non-obese patients. Patients with antegrade puncture had significantly shorter LoS than those with retrograde puncture (hazard ratio [HR] = 0.609 [95% CI: 0.486–0.827], *p* = 0.0085). Patients undergoing DUS-guided puncture had significantly shorter LoS than those without DUS (HR = 0.634 [95% CI: 0.486–0.827], *p* = 0.0008). Furthermore, TTH was significantly shorter with DUS (HR = 1.627 [95% CI: 1.247–2.122], *p* = 0.0003) than without it, although TTA was significantly longer with DUS than without DUS (HR = 1.681 [95% CI: 1.286–2.197], *p* = 0.0001).

## Discussion

FEMOSEAL CLOSE examined the safety and effectiveness of FemoSeal™ VCD in transfemoral percutaneous peripheral procedures in routine clinical practice. We observed high effectiveness and safety peri-procedurally, during hospital stay and up to 30 days after VCD deployment.

Most FemoSeal™ VCD studies included patients undergoing endovascular procedures for CAG [3, 4], with few data available for PAD endovascular revascularization. The STEP head-to-head trial compared FemoSeal™ with ProGlide™ in patients undergoing lower-limb arterial endovascular revascularization [5]. In this study, FemoSeal™ demonstrated technical success (defined as achievement of hemostasis without the need for a follow-up intervention at the access site and without a 2-g/dL drop in haemoglobin) in 80% of patients, significantly higher than achieved by ProGlide™ (50%; *p*< 0.0001) [5]. FEMOSEAL CLOSE demonstrated higher effectiveness, with 96.1% of patients achieving puncture-site haemostasis. The difference between these studies could be explained by different endpoint definitions. In fact, the STEP study did not distinguish types of bleeding such as small bruising, oozing, pulsatile bleeding, or the cause of an enlarging haematoma, which could overestimate failures, while the FEMOSEAL CLOSE protocol excluded oozing.

Herein, we report a low rate of device deployment failure (1.6%). However, device failure is often defined more broadly to include deployment failure and continuous bleeding despite successful deployment; this makes comparison between trials difficult. Using this definition, FemoSeal™ device failure rates of 6.4% and 5.3% in patients undergoing CAG have been reported in CLOSE-UP [3], and ISAAR-CLOSURE [4], respectively. The device failure rate for FemoSeal™ in the latter study was significantly lower than for ExoSeal^®^ VCD (Cordis, USA) (12%, *p*< 0.001) [4]. In the prospective registry of Angio-Seal™ (Terumo, Japan) and Perclose (Abbott, USA) VCDs, the failure rate was 2.7% [12]. In CLOSE-UP III, a randomised clinical study comparing MynxGrip™ (Cardinal Health, USA) VCD with MC, device failure was reported in 7% [13].

Low levels of combined major and minor complications were observed in FEMOSEAL CLOSE (8.7% at 30-day follow-up). In the STEP study, significantly fewer major access-site complications (defined as bleeding requiring MC, additional VCD, or another intervention; a 2 g/dL decrease in haemoglobin; or a pseudoaneurysm, local infection, acute ipsilateral leg ischaemia, or nerve injury related to VCD deployment) were reported for FemoSeal™ (20%) than ProGlide™ (56%, *p* < 0.0001) at 30-day follow-up [5]. The ISAAR-CLOSURE study reported a 6.0% rate of major and minor access-site complications with FemoSeal™ at 30 days in patients undergoing CAG [4]. However, such direct cross-study comparison is inappropriate due to differing endpoint definitions.

We noted a consistently low TTH with FemoSeal™ (0.42 min), which aligns with the TTH of 0.5 (range; 0.2–1.0) minutes reported previously [4]. By comparison, median TTH was 4 (range: 3–5) minutes for MynxGrip™ in patients undergoing CAG procedures [13]. A low TTH translates into ease of use for the study device. Indeed, physicians evaluated the study device as ‘easy’ or ‘very easy’ to deploy in 98.7% of cases.

Generally, using VCDs shortens the TTA compared with using MC [2]. The STEP study reported a mean duration of post-operative bed-rest (i.e. TTA) of 349 ± 78 min for the study device [5], which aligns with the median TTA of 5 h we report here. However, the TTA may have been shorter had all procedures been performed in outpatient settings. The German centre involved in our study mandates 6 hours’ bed-rest independent of VCD or MC-only use and this will have influenced the TTA results. Standard clinical practice regarding the duration of bed rest varies based on the hospital policy, the patient’s primary diagnosis, the complexity of the procedure, the patient’s overall condition, comorbidities, and the status of the access site. All these factors will influence the TTA. Hauguel et al. reported a wide variation in time to ambulation from 15 min to four hours [14]. However, any complications occurring after endovascular procedures tend to occur early (2–4 h), thus, a 4-hour post-operative observation period should catch most adverse effects [14]. The median LoS in this study was 23.98 h and was driven by a high rate of outpatient hospitalisation.

FEMOSEAL CLOSE also examined patient QoL, as better short-term QoL has been reported for VCDs versus MC in patients undergoing angiography [15]. Despite observing statistically significant improvements in QoL between pre-procedure and hospital discharge and from pre-procedure to 30-day follow-up, the overall number and size of the changes were small, making their clinical relevance unclear. Furthermore, the small changes may be explained by short intervals between measurements, and we cannot differentiate between QoL improvements related to the VCD and other aspects of the endovascular intervention.

The ISAAR-CLOSURE study previously reported that women required more MC than men and were at higher risk of vascular access-site complications [16]. We found no difference in any metric between men and women or between non-obese and obese patients.

An antegrade femoral approach, although routine for percutaneous treatment of lower extremities, is not included in VCD IFUs. We found no significant difference in TTH, TTA or complication rates between antegrade and retrograde puncture. These data support previous findings for the study device [17], and other reports of comparable haemostasis and safety for various VCDs employing both puncture types [18]. 

Ultrasonographic guidance of CFA access has been shown to decrease overall complication rate [19–23], especially groin haematoma [20], and may be useful in antegrade access and elderly patients [19]. Our subgroup analysis showed no difference in major and minor complications at 30 days between the DUS-guided and non-DUS-guided patients. However, the DUS-guided puncture group had significantly shorter TTH and LoS, although TTA was, paradoxically, significantly longer; the underlying reason for this remains unclear from clinical perspective.

As a single-arm study, outcomes can only be viewed in the context of historical cohorts not direct comparators. Such a comparison is often inaccurate, as endpoint definitions and study population are not consistent across datasets and publications.

## Conclusion

The FemoSeal™ VCD performs well in real-world settings and provides effective haemostasis and low rates of access-site complications for patients undergoing peripheral endovascular interventions by antegrade or retrograde CFA access. Its consistent effectiveness and safety across subpopulations, and good operator usability makes it a valuable and widely applicable device.

## Supplementary Information

Supplementary Material 1: Supplementary Figure. Distribution of change in EQ5D Health State Value (HSV).

## Data Availability

The data that support the findings of this study are available from Terumo Europe N.V. (study-sponsor) but restrictions apply to the availability of these data, which were used under license for the current study, and so are not publicly available. Data are however available from the corresponding author upon reasonable request and with permission of Terumo Europe N.V.

## Abbreviations

BARC: Bleeding Academic Research Consortium
BMI: Body mass index
CAG: Coronary angiography
CFA: Common femoral artery
CI: Confidence interval
DUS: Duplex ultrasound
FAS: Full analysis set
HR: Hazard ratio
IFU: Instructions for use
LoS: Length of stay
MC: Manual compression
PAD: Peripheral artery disease
QoL: Quality of life
SD: Standard deviation
TTA: Time to ambulation
TTH: Time to haemostasis
VCD: Vascular closure device

## References

[1] Reich R, Helal L, Mantovani VM, Rabelo-Silva ER. Hemostasis control after femoral percutaneous approach: a systematic review and meta-analysis. Int J Nurs Stud. 2023;137: 104364. 36399944 10.1016/j.ijnurstu.2022.104364

[2] Pang N, Gao J, Zhang B, et al. Vascular closure devices versus manual compression in cardiac interventional procedures: systematic review and meta-analysis. Cardiovasc Ther. 2022;2022:8569188. 36134143 10.1155/2022/8569188 PMC9482152

[3] Holm NR, Sindberg B, Schou M, et al. Randomised comparison of manual compression and FemoSeal™ vascular closure device for closure after femoral artery access coronary angiography: the CLOSure dEvices used in everyday practice (CLOSE-UP) study. EuroIntervention. 2014;10:183–90. 24603054 10.4244/EIJV10I2A31

[4] Schulz-Schüpke S, Helde S, Gewalt S, et al. Comparison of vascular closure devices vs manual compression after femoral artery puncture: the ISAR-CLOSURE randomized clinical trial. JAMA. 2014;312:1981–7. 25399273 10.1001/jama.2014.15305

[5] Gouëffic Y, Picquet J, Schneider F, et al. A randomized trial comparing polymer versus suture-based vascular closure devices for arterial closure following lower-limb arterial endovascular revascularization. Cardiovasc Intervent Radiol. 2021;44:1883–92. 34386892 10.1007/s00270-021-02940-z

[6] De Poli F, Leddet P, Couppie P, Daessle JM, Uhry S, Hanssen M. FemoSeal Evaluation Registry (FER). Prospective study of femoral arterial closure with a mechanical system on 100 patients who underwent angioplasty procedures. Ann Cardiol Angeiol (Paris). 2014;63:339–44. 25281993 10.1016/j.ancard.2014.08.009

[7] Wanitschek MM, Suessenbacher A, Dorler J, Pachinger O, Moes N, Alber HF. Safety and efficacy of femoral artery closure with the FemoSeal(R) device after coronary angiography using a 7 French sheath. Perfusion. 2011;26:447–52. 21712339 10.1177/0267659111409967

[8] Terumo Europe N.V. FemoSeal^™^ vascular closure system: instructions for use. 2022.

[9] Brooks R, Rabin R, De Charro F. The measurement and valuation of health status using EQ-5D: a European perspective: evidence from the EuroQol BIOMED Research Programme. Springer Science & Business Media; 2013. https://link.springer.com/book/10.1007/978-94-017-0233-1. 10.1007/978-94-017-0233-1.

[10] EuroQol Group 1. EuroQol--a new facility for the measurement of health-related quality of life. Health Policy. 1990;16(3):199–208. 10.1016/0168-8510(90)90421-9 10109801

[11] Brooks R. EuroQol: the current state of play. Health Policy. 1996;37:53–72. 10158943 10.1016/0168-8510(96)00822-6

[12] Bangalore S, Arora N, Resnic FS. Vascular closure device failure: frequency and implications: a propensity-matched analysis. Circ Cardiovasc Interv. 2009;2:549–56. 20031773 10.1161/CIRCINTERVENTIONS.109.877407 PMC3046770

[13] Jakobsen L, Holm NR, Maeng M, et al. Comparison of MynxGrip vascular closure device and manual compression for closure after femoral access angiography: a randomized controlled trial: the closure devices used in every day practice study, CLOSE-UP III trial. BMC Cardiovasc Disord. 2022;22:68. 35196986 10.1186/s12872-022-02512-0 PMC8864788

[14] Hauguel A, Maurel B, Bague N, et al. Management of ambulatory (day case) endovascular procedures for peripheral arterial disease. J Cardiovasc Surg. 2017;58(2):293–304. 28128542 10.23736/S0021-9509.17.09879-2

[15] Cox T, Blair L, Huntington C, Lincourt A, Sing R, Heniford BT. Systematic review of randomized controlled trials comparing manual compression to vascular closure devices for diagnostic and therapeutic arterial procedures. Surg Technol Int. 2015;27:32–44. 26680377

[16] Gewalt SM, Helde SM, Ibrahim T, et al. Comparison of vascular closure devices versus manual compression after femoral artery puncture in women. Circ Cardiovasc Interv. 2018;11:e006074. 30354782 10.1161/CIRCINTERVENTIONS.117.006074

[17] Tagliaferro FB, Orgera G, Mascagni L, et al. FemoSeal(^®^) vascular closure device for antegrade common femoral artery access: safety and technical notes. J Vasc Access. 2020;21:79–85. 31232151 10.1177/1129729819854593

[18] Barrette LX, Vance AZ, Mantell MP, Kratz KM, Redmond JW, Clark TWI. Safety and efficacy of arterial closure devices following antegrade femoral access: a case-control study. Vasc Endovascular Surg. 2020;54:612–7. 32721190 10.1177/1538574420941298

[19] Fukuda K, Okazaki S, Shiozaki M, et al. Ultrasound-guided puncture reduces bleeding-associated complications, regardless of calcified plaque, after endovascular treatment of femoropopliteal lesions, especially using the antegrade procedure: a single-center study. PLoS One. 2021;16: e0248416. 33711058 10.1371/journal.pone.0248416 PMC7954350

[20] Kalish J, Eslami M, Gillespie D, et al. Routine use of ultrasound guidance in femoral arterial access for peripheral vascular intervention decreases groin hematoma rates. J Vasc Surg. 2015;61:1231–8. 25595399 10.1016/j.jvs.2014.12.003

[21] Lo RC, Fokkema MT, Curran T, et al. Routine use of ultrasound-guided access reduces access site-related complications after lower extremity percutaneous revascularization. J Vasc Surg. 2015;61:405–12. 25240244 10.1016/j.jvs.2014.07.099 PMC4308537

[22] Lucatelli P, Cannavale A, Cirelli C, d’Adamo A, Salvatori FM, Fanelli F. Use of ultrasound in the insertion of a vascular closure device: a comparative retrospective study with the standard blind technique. Radiol Med. 2015;120:283–8. 25120078 10.1007/s11547-014-0439-3

[23] Rashid MK, Sahami N, Singh K, Winter J, Sheth T, Jolly SS. Ultrasound guidance in femoral artery catheterization: a systematic review and a meta-analysis of randomized controlled trials. J Invasive Cardiol. 2019;31:E192-198. 31257213 10.25270/jic/19.00090

