# Supplementary material for: FEMOSEAL CLOSE: multi-centre observational study with FemoSeal™ vascular closure device following peripheral percutaneous endovascular procedures
Source: CVIR Endovasc. 2025 Feb 22;8:15. doi: 10.1186/s42155-025-00522-5 (PMC11846783; doi:10.1186/s42155-025-00522-5)
Supplement: Supplementary file 1 — Supplementary Material 1: Supplementary Figure. Distribution of change in EQ5D Health State Value (HSV). [file 42155_2025_522_MOESM1_ESM.docx]

***Supplementary Figure: Distribution of change in EQ5D Health State Value (HSV)***
